# Ghrelin, food intake, and botanical extracts: A Review

**Published:** 2015

**Authors:** Peyman Rezaie, Mohsen Mazidi, Mohsen Nematy

**Affiliations:** 1 *Biochemistry of Nutrition Research Center, School of Medicine, Mashhad University of Medical Sciences, Mashhad, Iran*; 2 *Institute of Genetics and Developmental Biology, International College, University of Chinese Academy of Science (IC-UCAS), Beijing, China*

**Keywords:** *Ghrelin*, *Food intake*, *Botanical extracts*, *Appetite*

## Abstract

A kind of growth hormone secretagogue (GHS), ghrelin, was first isolated from the rat stomach and plays a major role in the activation of the growth hormone secretagogue receptor 1a (GHS-R1a) resulting the release of growth hormone (GH). The preproghrelin gene is placed on chromosome 3, at locus 3p25 –2 in humans and constitutes five exons and three introns. Ghrelin is most plentifully expressed in particular cells in the oxyntic glands of the gastric epithelium, initially named X/A-like cells. Almost 60-70% of circulating ghrelin is secreted by the stomach. Plasma ghrelin concentration alters throughout the day. Ghrelin has been suggested to act as a meal initiator because of its appetite-stimulating influences in free feeding rats in short period. In addition to ghrelin’s function as a meal motivator, it seems to contribute in long-term energy balance and nutritional status. In addition, many studies have been carried out in order to investigate the effects of natural and medicinal plants and botanical extracts on appetite, food intake, energy hemostasis, and the level of related hormones including ghrelin. Due to the importance of ghrelin in nutritional and medical sciences, this review was performed to understand new aspects of this hormone’s function.

## Introduction

A kind of growth hormone secretagogue (GHS), ghrelin, was first isolated from the rat stomach in 1999 by Kojima and colleagues (Kojima et al., 1999 ▶, Khatib et al., 2014 ▶). The name “ghrelin” is derived from “ghre” which means grow and “relin” which means release (Walker et al., 2013 ▶). GHSs perform through the GHS-receptor (GHS-R) which is a G protein-coupled receptor (GPCR) and plays a major role in the activation of the growth hormone secretagogue receptor 1a (GHS-R1a) resulting the release of growth hormone (GH) (Seim et al., 2007 ▶, Laviano et al., 2012 ▶, Kojima and Kangawa, 2005 ▶, Kojima et al., 1999 ▶, Abu-Farha and Dehbi, 2014 ▶).

The ghrelin receptor (GHS-R1a) is preserved across dissimilar vertebrate species of mammals, birds, and fishes. Transcripts for GHS-R1A are expressed at low levels in many tissues, however mainly in the arcuate and ventromedial nuclei of the hypothalamus, pituitary hippocampus in addition to low-level expression in kidney, pancreas, liver, lung, heart, gastrointestinal tract, adipose tissue, and immune cells (Walker et al., 2013 ▶, Seim et al., 2007 ▶, Laviano et al., 2012 ▶, Kojima and Kangawa, 2005 ▶, Khatib et al., 2014 ▶, Abu-Farha and Dehbi, 2014 ▶). The finding of ghrelin has increased comprehending of the relation between the stomach and the brain and has shed new light on several physiologic procedures, comprising gastrointestinal activity, the adjustment of pituitary hormone secretion, and energy homeostasis as well as cardiovascular function (Wren et al., 2001 ▶, Ukkola, 2011 ▶, Tritos and Kokkotou, 2006 ▶, Scerif et al., 2011 ▶, Kojima et al., 2001 ▶, Kojima et al., 2004 ▶, Kojima et al., 1999 ▶, Isgaard and Granata, 2011 ▶).

**Ghrelin gene**

The preproghrelin gene is placed on chromosome 3, at locus 3p25 –2 in humans and constitutes five exons and three introns (Seim et al., 2007 ▶). Ghrelin gene shows polymorphism and is not connected with any variations in circulating ghrelin concentration (Vivenza et al., 2004 ▶). Spliced ghrelin messenger RNA is translated to a 117- amino acid preproghrelin precursor, which is consequently cleaved to final ghrelin with 28-amino-acid peptide (Nematy et al., 2006 ▶, Koleva et al., 2013 ▶). Ghrelin has dissimilar isoforms containing acylated form at Ser with a fatty acid or has a deletion of the C-terminal arginine at position 28 which is made by enzymatic process (Seim et al., 2007 ▶). Moreover, there is also a nonacylated ghrelin form (des-acyl ghrelin) which exists in excess in plasma with unclear physiologic role (Hosoda et al., 2000 ▶, Date et al., 2000 ▶, Cao et al., 2013 ▶). 

**Ghrelin expression**

Ghrelin is most abundantly expressed in particular cells in the oxyntic glands of the gastric epithelium, initially named X/A-like cells (Tritos and Kokkotou, 2006 ▶, Date et al., 2000 ▶). Almost 60-70% of circulating ghrelin is secreted by the stomach, and furthermost of the remainder emanates from the small intestine (Tritos and Kokkotou, 2006 ▶, Date et al., 2000 ▶). Moreover, ghrelin is made in small quantities by other organs such as lung, heart, kidney, lymphatic tissue, adrenal glands, pancreas, thyroid gland, some neoplastic tissues, and cancer-cell lines (Wierup et al., 2004 ▶, Prado et al., 2004 ▶, Miki et al., 2012 ▶, Lu et al., 2002 ▶, Kim et al., 2012 ▶, Khatib et al., 2014 ▶, Fukushima et al., 2005 ▶, Cowley et al., 2003 ▶, Cao et al., 2013 ▶).Various investigations also concluded that ghrelin immunoreactivity originates from the testis, including both Sertoli and Leydig cells and the placenta (Tritos and Kokkotou, 2006 ▶, Tena-Sempere et al., 2002 ▶, Gualillo et al., 2001 ▶, Barreiro et al., 2002 ▶) as well as low ghrelin concentrations in the cerebrospinal fluid with uncertain origin (Tschop et al., 2001 ▶, Tritos and Kokkotou, 2006 ▶, Tritos et al., 2003 ▶). As mentioned before, ghrelin exists in placenta along GH, GHRH, and insulin-like growth factor 1 (IGF1) which demonstrates that it also plays a role in fetal growth (Martin et al., 2011 ▶, Khatib et al., 2014 ▶, Emanuel and Ritter, 2010 ▶). Ghrelin is highly preserved between mammals and has even been identified in chickens, bullfrogs, and fish which indicates its significant evolutionary position (Tritos and Kokkotou, 2006 ▶, Kojima et al., 1999 ▶, Emanuel and Ritter, 2010 ▶).

**Ghrelin level**

Plasma ghrelin concentration alters throughout the day. Its level is maximum in the fasting state, before meals, and at night, falling within one hour of a meal (Martin et al., 2011 ▶, Cummings et al., 2001 ▶, Bednarek et al., 2000 ▶) particularly by high-calorie or high-carbohydrate meals (Tritos and Kokkotou, 2006 ▶, Nematy et al., 2007 ▶, Dardzinska et al., 2014 ▶, Cummings et al., 2002 ▶). Furthermore, evidences indicated that the consumption of a low-ED (energy dense) diet for weight loss is related with lower circulating level of ghrelin, which may assist in weight loss or maintenance (Weigle et al., 2003 ▶, Hill et al., 2013 ▶). Fat seems to decrease ghrelin less effectively per calorie than carbohydrate or protein. This may elucidate the reduced satiety and increased weight gain connected with high-fat diets to some extent (Wren and Bloom, 2007 ▶, Overduin et al., 2005 ▶, Monteleone et al., 2003 ▶).

The association between pre-prandial rise in ghrelin in humans with hunger scores indicates that ghrelin plays as a meal-initiation signal in the short-term adjustment of appetite (le Roux et al., 2005 ▶, Klok et al., 2007 ▶). Weight loss, fasting, and hypoglycemia augment ghrelin mRNA expression and secretion (Howe et al., 2014 ▶, Coll et al., 2007 ▶), however oral or intravenous glucose, glucagon, insulin, somatostatin, and GH decrease systemic ghrelin concentration (Tritos and Kokkotou, 2006 ▶, Qi et al., 2003 ▶, Norrelund et al., 2002 ▶, Nematy et al., 2006 ▶, Klok et al., 2007 ▶, Howe et al., 2014 ▶, Coll et al., 2007 ▶, Barkan et al., 2003 ▶, Arafat et al., 2005 ▶). Moreover, investigations reported that ghrelin plasma level decreases due to some diseases including untreated hyperthyroidism, in the presence of *Helicobacter pylori–*induced gastritis or after major surgery such as total gastrectomy, gastric bypass surgery, and coronary artery bypass grafting (Takachi et al., 2006 ▶, Nematy et al., 2007 ▶, Isomoto et al., 2005 ▶). Nematy et al. reported high levels of PYY and low levels of ghrelin in ICU patients compared to healthy controls (Nematy et al., 2006 ▶). Some studies concluded that circulation level of ghrelin increases during some diseases including Prader-Willi syndrome and cardiac diseases and is associated with malignancy which induces cachexia and anorexia nervosa (Tritos and Kokkotou, 2006 ▶, Rojdmark et al., 2005 ▶, Riis et al., 2003 ▶).

Short-term infusions of oxyntomodulin, urocortin, peptide YY, and all recognized appetite-suppressing peptides cause a decrease in plasma ghrelin levels (Davis et al., 2004 ▶). In contrast, leptin administration does not seem to have an influence on plasma ghrelin level (Chan et al., 2004 ▶).

Overall, it should be noted that factors involved in the regulation of systemic ghrelin levels and the underlying procedures have not been completely studied and several fields of debates still exist.

**Ghrelin and appetite**

Ghrelin has been suggested to act as a meal initiator because of its appetite-stimulating influences in free feeding rats in short period (Tschop et al., 2000 ▶, Perry and Wang, 2012 ▶). Ghrelin has also been indicated to induce appetite as effectively as NPY, formerly the most influential recognized orexigen, in both lean and obese humans (Wren et al., 2001 ▶, Perry and Wang, 2012 ▶, Neary et al., 2004 ▶, Nakazato et al., 2001 ▶, Druce et al., 2005 ▶). Subcutaneous ghrelin administration causes an increase in appetite and food intake in obese and lean humans (Macke et al., 2009 ▶, Druce et al., 2005 ▶). Fasting ghrelin levels in obese subjects have been stated to be lower compared to normal weight individuals and increase following diet-induced weight loss (Perry and Wang, 2012 ▶, Nakazato et al., 2001 ▶, Cummings et al., 2002 ▶). The role of ghrelin in pathogenesis of obesity can be inferred due to attenuated suppression of postprandial serum ghrelin in obese individuals (le Roux et al., 2005 ▶, English et al., 2002 ▶, Cummings et al., 2002 ▶).

**Ghrelin and energy hemostasis**

In addition to ghrelin’s function as a meal motivator, it seems to contribute to long-term energy balance and nutritional status in the reverse way to leptin. Systemic ghrelin amounts are adversely connected with body adiposity, being low in the obese and higher in lean subjects.

It has also been indicated that obese subjects may be more sensitive to appetite motivation by exogenous ghrelin. Therefore, when ghrelin level raises, inhibition of ghrelin secretion may have therapeutic potential, generally in enhancing further weight loss and inhibiting weight regain after diet-induced weight loss (Wren and Bloom, 2007 ▶, Druce et al., 2005 ▶). Ghrelin level is decreased by overfeeding, prosperous treatment of anorexia nervosa, glucocorticoid administration, and weight gain induced by high-fat diet (Wren and Bloom, 2007 ▶, Robertson et al., 2004 ▶, Hansen et al., 2002 ▶).

Furthermore, this hormone is increased by exercise, weight loss caused by low-calorie diet, anorexia nervosa, or cachexia as a result of organ failure such as cardiac, pulmonary, renal, or hepatic or some type of malignancy (Tritos and Kokkotou, 2006 ▶, Otto et al., 2004b ▶, Otto et al., 2005 ▶, Otto et al., 2004a ▶, Otto et al., 2001 ▶). Moreover, it has been revealed that chronic administration of ghrelin in rodents results in prolonged hyperphagia and weight gain (Wren and Bloom, 2007 ▶, Tschop et al., 2001 ▶). As well, chronic and long term administration of ghrelin cause activation of adipogenesis, inhibition of apoptosis, transmission from fatty acid oxidation to glycolysis for energy expenditure, inhibition of sympathetic nervous system activity, and reduce energy expenditure and unplanned activity (van der Lely et al., 2004 ▶, Thompson et al., 2004 ▶, Nakazato et al., 2001 ▶, Matsumura et al., 2002 ▶, Choi et al., 2003 ▶).

**Ghrelin, food intake and botanical extracts**

Recently, many studies have been carried out in order to investigate the effects of natural and medicinal plants and botanical extracts on appetite, food intake, energy hemostasis, and the level of related hormones including ghrelin. This review was performed to highlight some of the related studies in this field and to find theie importance in nutritional sciences and new insight of using these plants in clinical approaches in parallel to prescribed medication especially cyproheptadine hydrochloride which is the most widely prescribed medication for anorexia which possesses several side effects.

Mazidi et al. carried out an investigation in order to study the effect of hydroalcoholic extract of Cannabis sativa on appetite hormone in male Wistar rats (Mazidi et al., 2014a ▶). Cannabis sativa is recognized as an orexigenic herb in Iranian traditional medicine. Little evidences are available about its influence on energy intake and its mechanism (Riggs et al., 2012 ▶, Fride et al., 2005 ▶). Their results showed that the extract of cannabis sativa meaningfully increased energy intake and total ghrelin levels were significantly increased in the Cannabis sativa group. Their investigation revealed both positive and dose-related impacts of cannabis sativa on appetite of rats (Mazidi et al., 2014a ▶).

 Moreover, some studies concluded the appetite-stimulating effects of the cannabis plant and its role through two cannabinoid receptors in brain and peripheral organ systems and endogenous ligands (endocannabinoids) for these receptors in human body as well as its new vital clinical role in improving the general health condition and food intake by some critically ill patients such as patients with HIV (Human Immunodeficiency Virus) or cancer (Waissengrin et al., 2014 ▶, Sansone and Sansone, 2014 ▶, Ruchlemer et al., 2014 ▶, Kirkham and Williams, 2001 ▶, Kirkham, 2005 ▶, Gazdek, 2014 ▶).

In addition, it has been published that extract of *Hoodia gordoni* is one of the most popular herbal supplements that possesses appetite suppressant properties (Rayalam et al., 2008 ▶, Avula et al., 2006 ▶). This extract adjusts food intake and has an adverse effect on ghrelin level (Avula et al., 2006 ▶). Several other herbal supplements and plant extracts such as ephedra, Citrus aurantium, hydroxycitric acid, and Phaseolus vulgaris isolectins have also been stated to have appetite-suppressing properties (Ohia et al., 2002 ▶, Kuriyan et al., 2007 ▶, Klontz et al., 2006 ▶, Fleming, 2007 ▶).

Sengupta et al. conducted a study in order to investigate the efficacy of Dolichos biflorus and Piper betle extracts on weight management. Their results showed that Piper betle leaf extract and Dolichos biflorus seed extract have potent anti-adipogenic efficacy (Sengupta et al., 2012 ▶). In this study, declines in body weight and BMI were detected after 8 weeks of supplementation, in addition meaningful increase in serum adiponectin concentration and significant decline in serum ghrelin concentration were observed.

Some studies examined the role of arabinoxylan in regulating ghrelin secretion. Arabinoxylan is the main dietary fibre type in whole-grain rye and constitutes 8-9% of its dry weight (Vinkx and Delcour, 1996 ▶, Isaksson et al., 2009 ▶). Studies reported that when 15 g/day of wheat arabinoxylan was consumed during a 6-wks period by contributors with impaired glucose tolerance, total plasma ghrelin concentrations (4 h postprandial) were dropped at the end of the period (Garcia et al., 2007 ▶).

Mazidi and his colleagues carried out an investigation in order to study the influences of hydroalcoholic extract of Coriandrum sativum on ghrelin hormone in rats (Mazidi et al., 2014b ▶). *Coriandrum sativum* (coriander) is a herb belonging to the family Apiaceous. It is digestive and appetite stimulating in traditional medicine (Emamghoreishi et al., 2005 ▶). The results of this study suggested that *Coriandrum sativum* has no effect on the level of the ghrelin hormone.

Moreover, Nematy et al. investigated the effect of hydroalcoholic extract of Coriandrum sativum on thirty male Wistar rats’ appetite (Nematy et al., 2013 ▶). In this study, the daily amount of the food consumed by each rat was determined for 10 days and the amount of energy intake of each rat was also measured for 7 days throughout the intervention. Their results indicated that coriander has positive impacts on appetite of rats.

Shen et al. demonstrated that food intake was enhanced by linalool owing to decrease in plasma glycerol levels; consequently it causes an elevation in body weight (Shen et al., 2005 ▶). Since, the linalool is one of the constituents of *Coriandrum Sativum *essential oils (Usta et al., 2009 ▶), it can also be a proposed mechanism for the orexigenic influence of this plant.

Cornelian Cherry (*Cornus mas L*.) is another medicinal plant with various functional aspects in traditional medicine. Narimani-Rad et al. conducted an investigation to study the effect of Cornelian Cherry (*Cornus mas L*.) extract on serum ghrelin and corticosterone levels in rats (Narimani-Rad et al., 2013 ▶). Their results revealed that infusion of cornelian cherry fruit extract in different quantities has not any effect on ghrelin and corticosteroid secretion. Though, it may have substantial impact on glycemic status.

Ginger (*Zingiber officinale*) is another herbal medicine which has been used to treat a number of medical conditions, including nausea, dyspepsia, flatulence, abdominal pain, and improvement in food intake and digestion (Cupp, 2000 ▶, Hu et al., 2011 ▶). However, the mechanisms accountable for its advantageous effects are not well recognized. Studies reported that its action on gastric motility and improvement in food intake could be mediated through augmented secretion of ghrelin or motilinor through suppression of glucagon like peptide-1 (Tack et al., 2006 ▶, Luiking et al., 2003 ▶).

Furthermore, Muhammad S. Mansouret al. carried out a study to assess the effects of ginger consumption on hormonal parameters in overweight men. They concluded that total ghrelin levels were significantly developed after ginger consumption compared to the no ginger condition, with the major difference happening at 45 min after consumption (Mansour et al., 2012 ▶). Results of other studies are in accordance with this outcome (Ueki et al., 2008 ▶, Drazen et al., 2006 ▶).

It can be inferred that the discovery of ghrelin has provided novel and unique information on the interrelationships between stomach and brain and expands our understanding of the regulation of GH secretion, energy homeostasis, and food intake. Ghrelin plays a vital role in improving the health condition through its part in stimulating appetite and providing adequate energy intake in some of the hospitalized patients especially critically ill patient such as ICU patients and patients with major surgery, cancer, or HIV. As it is mentioned in this review, some of the botanical extracts and medicinal plants have been suggested to affect the ghrelin level in human body; consequently regulate the feel of appetite and satiety without any critical side effects compared to prescribed medications particularly in ill patients or underweight and overweight individuals. It can be concluded that there is a new insight of using traditional medicinal plants in medical sciences mainly in nutritional fields in order to improve and treat some of the diseases. However, more comprehensive investigations are required in order to reach a reasonable conclusion about this field of study.
